# Seroprevalence and Association of *Toxoplasma gondii* with Bone Health in a Cohort of Osteopenia and Osteoporosis Patients

**DOI:** 10.3390/biomedicines12071400

**Published:** 2024-06-24

**Authors:** Indulekha Karunakaran, Jayagopi Surendar, Pia Ransmann, Marius Brühl, Silvia Kowalski, Victoria Frische, Jamil Hmida, Sabine Nachtsheim, Achim Hoerauf, Dieter C. Wirtz, Marc P. Hübner, Andreas C. Strauss, Frank A. Schildberg

**Affiliations:** 1 Institute for Medical Microbiology, Immunology and Parasitology, University Hospital Bonn, 53127 Bonn, Germany; 2 Department of Orthopedics and Trauma Surgery, University Hospital Bonn, 53127 Bonn, Germany; 3 German Center for Infection Research (DZIF), Partner Site Bonn-Cologne, 53127 Bonn, Germany

**Keywords:** osteopenia, osteoporosis, bone health, *Toxoplasma gondii*, seroprevalence, musculoskeletal disease

## Abstract

Considering the fact that *Toxoplasma* is a common parasite of humans and *Toxoplasma* bradyzoites can reside in skeletal muscle, *T. gondii*-mediated immune responses may modulate the progression and pathophysiology of another musculoskeletal disorder, osteoporosis. In the current study, we investigated the association of bone health and *Toxoplasma gondii* infection status. A total of 138 patients living in Germany with either osteopenia or osteoporosis were included in the study, and they were categorized into two groups, *T. gondii* uninfected (*n* = 74) and infected (*n* = 64), based on the presence of *T. gondii*-specific IgG antibodies. The demographic and clinical details of the study subjects were collected from the medical records. Logistic regression analysis was performed to delineate the association of bone health parameters with the infection status. The prevalence of toxoplasmosis was 46.4% in the study participants. The infected individuals with osteopenia and osteoporosis showed higher levels of mean spine and femoral T score, Z score, and bone mineral density (BMD), indicating improved bone health compared to the uninfected group. Logistic regression analysis showed that subjects with *T. gondii* infection displayed increased odds of having a higher mean femur T score, femur BMD, and femur Z score even after adjusting for age, creatinine, and urea levels. However, when the duration of drug intake for osteoporosis was taken into account, the association lost statistical significance. In summary, in this study, an improvement in osteopenia and osteoporosis was observed in *Toxoplasma*-infected patients, which may be partly due to the longer duration of drug intake for osteoporosis in the infected patient group.

## 1. Introduction

Traditionally regarded as an age-related endocrine (especially estrogen deficiency-mediated) disorder, osteoporosis is characterized by a systemic impairment of bone mass and microarchitecture that results in fragility fractures [1]. Affecting one in three women and one in five men at the age of 50 worldwide [2,3,4], as well as a total of 7.8 million people in Germany [5], it is one of the most underdiagnosed and undertreated diseases owing to its clinically silent nature before it clinically manifests as a fracture [5]. Fractures are the result of disproportionate bone formation and bone resorption owing to an imbalance in a well-balanced physiological process known as “bone remodeling” [6]. In addition, recent advances in the field of osteoimmunology had led to the identification of the disease as an inflammatory disorder [7]. With increasing evidence pointing to the cross-talk between the skeletal and the immune system, the impact of “inflammaging”—chronic low-grade inflammation in aging—on the pathophysiology of osteoporosis is increasingly being delineated [8].

Capable of infecting all warm-blooded animals, the protozoan parasite *Toxoplasma gondii* is prevalent in 30% of the world’s population [9]. With marked global variability in seroprevalence, ranging from 10 to 90%, there are endemic hotspots in almost all the continents—the USA, Europe, Latin America, Asia, and Australia [10]. In Europe alone, its prevalence ranges from 10 to 60% [10], with Germany showing 50% seroprevalence among adults, which increases with increasing age [11]. Transmitted primarily through felines, which are the only known definitive hosts, the parasite is shed as oocysts through the feces of domestic cats and felines [12] and contaminates crops, soil, and water resources [12]. Another route of transmission is through the bradyzoites, a dormant tissue resident form, which are ingested through the consumption of raw or undercooked meat [13,14]. Lastly, congenital toxoplasmosis is an acknowledged public health problem [15], as vertical transmission from mother to fetus (third possible route of infection) can result in serious postnatal abnormalities like chorioretinitis, intracranial calcifications, hydrocephalus, and learning deficits in early or later life [16]. Therefore, a major focus of interventions targeting toxoplasmosis has been to prevent congenital toxoplasmosis. However, the substantial burden of toxoplasmosis occurring as sporadic outbreaks of acute symptomatic disease in immunocompetent individuals has received little attention so far [17]. Of greater importance is the severe toxoplasmosis occurring in immunocompromised individuals like HIV patients, patients on immunosuppressive agents, etc., leading to toxoplasmic encephalitis [18] or visual impairments. These severe forms of toxoplasmosis can predominantly occur after reactivation of a chronic, latent infection in immunocompromised patients, which is of importance, as *T. gondii* infection is lifelong. With immunosuppressive agents and anti-inflammatory drugs becoming the norm for treatment in a range of age-related inflammatory and endocrine disorders like osteoporosis, it could be hypothesized that these disorders could be permissive for *T. gondii,* especially in a setting with high seroprevalence. Further, immunity against the parasite mediated by IL-12 and IFN-γ release combined with parasite-induced immune cell invasion and host manipulation might warrant damaging consequences for osteoporosis progression in the form of increased inflammation.

Our group has shown in the past that helminth infections exert profound immunomodulatory effects on the host, resulting in altered disease outcomes in case of autoimmune and metabolic diseases. We have demonstrated that helminth-induced immunomodulation could improve sepsis [19], type 1 diabetes [20], and diet-induced insulin resistance [21] mediated by a predominant Th2 response. Considering the fact that *Toxoplasma* is prevalent in a substantial proportion of the German population, as well as the residence of the bradyzoites in the skeletal muscle, the possibility of the *T. gondii*-mediated immune response modulating the progression and pathophysiology of another musculoskeletal disorder, osteoporosis, has never been explored. Further, the question of whether inflammaging in osteoporosis patients and intake of immunosuppressive drugs/hormone therapy could serve as a permissive factor for the reactivation of the dormant bradyzoites into disseminating tachyzoites has not been addressed in this context. Here, in this study, we hypothesize that *T. gondii* infection could modulate the pathology of osteoporosis. Drawing on our previous experience with helminth-mediated immunomodulation in autoimmune and metabolic diseases, we plan to extend this theme to address the association between *T. gondii* infection and osteoporosis in humans. This is a case–control study conducted in a risk population comprising osteopenia and osteoporosis subjects. *Toxoplasma* screening was conducted in the study subjects, and the association of toxoplasmosis with bone health indices of the spine and femur was delineated.

## 2. Materials and Methods

### 2.1. Study Subjects

Subjects were recruited during orthopedic consultation hours at the University Hospital of Bonn, Germany, between 2021 and 2023. The study included a total of 200 patients with low bone mineral density. A total of 138 of these individuals met the inclusion criteria for the investigation. According to the WHO criteria, 38.4% (*n* = 53) of the patients were identified as having osteopenia, whereas the remaining patients were diagnosed with osteoporosis (61.6%, *n* = 85). The exclusion criteria encompassed those with hepatitis and/or HIV infection, cancer, or transplantation. In addition, the study excluded patients having a history of urolithiasis, liver cirrhosis, congestive heart failure, chronic lung disorders, or any known renal diseases. All included patients were immunocompetent. Each study subject provided their informed consent by signing a consent form. Past and current osteoporosis-related medications (Denosumab, Romosozumab, Teriparatid, Bisphosphonate) were documented. The study was authorized by the institutional ethical committee of the University Hospital Bonn (ID: 283/21).

### 2.2. Dual X-ray Absorptiometry

Following the German osteoporosis guidelines, all patients underwent a dual X-ray absorptiometry (DXA) using Horizon^TM^ (Hologic, Marlborough, MA, USA) to assess the spine (vertebrae bodies 1–4) and the left femoral neck [22]. In case of arthroplasty of the left hip or a non-evaluable spine, the right hip or lower arm was examined in agreement with the guidelines [22]. The precise values of bone mineral density (BMD) in g/cm^2^ as well as T scores and Z scores were recorded. The T score indicates the standard deviation from the average BMD of a healthy young population of the same ethnicity, about 30 years old. The Z score compares the BMD results with the average BMD of individuals of the same age. According to the World Health Organization (WHO), using the lowest T score from either the femoral neck or spine (the sum of vertebrae bodies 1–4), the patient can be classified into the following three categories: normal (T score ≥ −1.0), osteopenia (T score ranging from −1.0 to −2.4), or osteoporosis (T score ≤ −2.5) [2,22].

### 2.3. Biochemical Parameters Measurement

All the biochemical parameters were measured using autoanalyzers. Enzymes including alkaline phosphatase, alanine-aminotransferase, aspartate-aminotransferase, creatinine kinase, gamma-glutamyltransferase, and lactate dehydrogenase were quantified using visible light (VIS) photometry. Bone alkaline phosphatase was quantified using spectrophotometry. The electrochemiluminescent immunoassay was utilized to quantify thyroid-stimulating hormone (TSH). The nutrients calcium, magnesium, inorganic phosphate, and iron were quantified using VIS photometry (Roche Cobas 8000, Roche Diagnostics, Mannheim, Germany). Sodium and potassium were measured using indirect potentiometry. Ferritin was examined using an electrochemiluminescent immunoassay, whereas 25-hydroxy vitamin D was measured using liquid chromatography–mass spectrometry. We utilized VIS photometry to quantify kidney function markers, including total bilirubin, urea, and creatinine. An immunoassay utilizing turbidimetry was employed to quantify immunoglobulins A, G, and M. Leucocytes and CRP levels were assessed by flow cytometry and turbidimetric immunoassay, respectively.

### 2.4. Toxoplasma Infection Screening, IgG and Avidity

Serum samples from the study participants were measured for *T. gondii*-specific IgG levels using the BioMérieux mini Vidas with the respective Vidas Toxo IgG II kit (BioMérieux, Marcy-l’Étoile, France). Patient samples that were positive for *T. gondii*-specific IgG were subsequently tested for *T. gondii*-specific IgG avidity (Vidas Toxo IgG Avidity, BioMérieux). Samples showing low IgG avidity were additionally tested for IgM (Vidas Toxo IgM, BioMérieux) to identify acute *Toxoplasma* infections. None of the tested participants had an acute infection.

### 2.5. Statistical Analysis

The analysis was conducted using GraphPad Prism v 9.4.1 and SPSS (v 24; IBM, Armonk, NY, USA). For continuous variables that exhibited normal distribution, Student’s *t* test was employed to compare groups, whereas the Mann–Whitney U test was utilized for continuous variables that did not conform to a normal distribution. A chi-square test was employed to compare the proportions between different groups. In order to assess the impact of different risk factors on the relationship between *Toxoplasma* infection and bone health, a regression analysis was conducted. Regression analysis is a statistical method used to investigate the relationship between two different variables. The uniqueness of this method is that it can control the association for numerous confounders. *Toxoplasma* infection was treated as the dependent variable, while the mean T score, Z score, and BMD were considered as the independent variables. Initially, a univariate analysis was conducted and adjusted models were constructed using multivariate regression analysis to determine the association of *Toxoplasma* infection with bone health by adjusting for other variables, like age, urea, creatinine, and duration of the therapy. We conducted a Pearson correlation analysis to determine the relationship between femoral and spinal bone health parameters and *T. gondii*-specific IgG levels and IgG avidity.

## 3. Results

In the total study population consisting of osteopenia (38.4%, *n* = 53) and osteoporosis (61.6%, *n* = 85) subjects, the prevalence of *Toxoplasma* was 46.4% (*n* = 64). All of the *Toxoplasma*-infected individuals had a chronic *Toxoplasma* infection, as indicated by the IgG and IgG avidity profile (Table 1).

**Figure 1:**
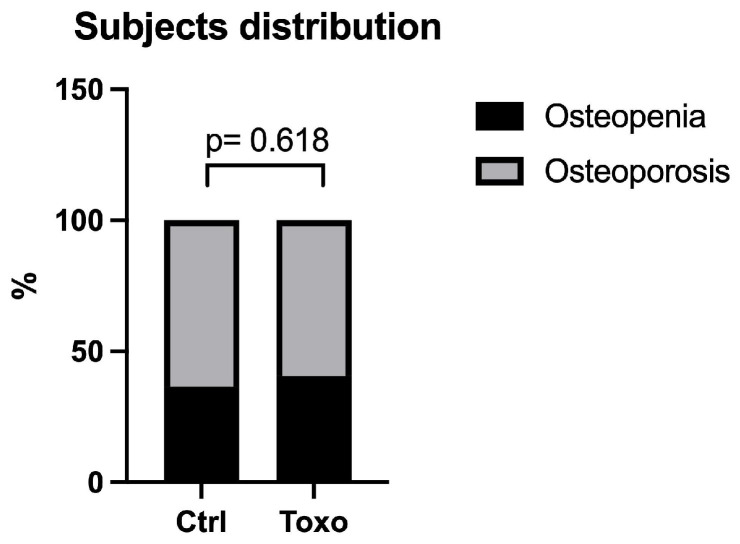
Distributions of study subjects in the uninfected (Ctrl) and infected (Toxo) groups. Percentage of osteopenia and osteoporotic study subjects in the study groups. A chi-square test was used to compare proportions between groups.

For further analyses, we split the study group into uninfected (*n* = 74) and infected subjects (*n* = 64). As shown in Figure 1, the distributions of subjects with osteopenia and osteoporosis in the uninfected and infected groups were similar (*p* = 0.618).

Table 1 shows the clinical and biochemical characteristics of the study subjects. The infected group, comprising osteoporotic and osteopenic patients positive for *Toxoplasma,* was significantly older (uninfected: 65.9 ± 10.2 vs. infected: 74.2 ± 9.6 years) compared to the uninfected group, which included osteoporotic and osteopenic subjects negative for toxoplasmosis. Further, osteoporotic subjects with *Toxoplasma* had significantly lower levels of vitamin D (45.6 ± 17.1 vs. 38.6 ± 17.1 ng/mL) and higher urea (30.3 ± 8.2 vs. 34.6+ ± 12.5 mg/dL) and creatinine levels (0.77 ± 0.19 vs. 0.86 ± 0.31 mL/min) compared to the control subjects negative for toxoplasmosis. However, the levels of micronutrients, as exemplified by calcium, magnesium, sodium, potassium, iron, and ferritin, were not different between the two groups. The incidence of fractures, as shown by the fracture history, was higher in the *Toxoplasma*-positive patients compared to the uninfected group (55.4% vs. 78.1%). The prevalences of other diseases, like COPD, Crohn’s disease, and rheumatoid arthritis, were not different between the two groups, and the medication details were also similar between the groups. However, the infected group showed a longer duration of drug intake for osteoporosis (5.1 ± 3.6 vs. 7.4 ± 5.2 years). The average level of the inflammatory marker CRP was higher in the *Toxoplasma* group according to the trend (4.7 ± 9.1 vs. 9.8 ± 18.3 mg/L), but the trend did not reach statistical significance. The levels of alkaline phosphatase, alanine aminotransferase, aspartate aminotransferase, creatinine kinase, gamma-glutamyltransferase, lactate dehydrogenase, and thyroid-stimulating hormone were also not different between the uninfected and *Toxoplasma*-positive group.

**Figure 2:**
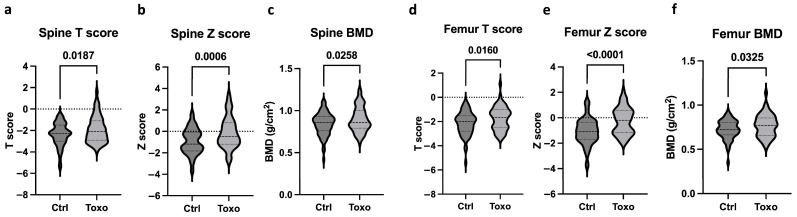
Bone health indices in the uninfected (Ctrl) and infected (Toxo) subjects. The violin plots depict the mean spinal T score (**a**), Z score (**b**), and BMD (**c**). The violin plots show the mean femoral T score (**d**), Z score (**e**), and BMD (**f**). Statistical significance was determined.

Figure 2 shows the bone health parameters between the uninfected subjects and *Toxoplasma*-infected individuals. The infected individuals with osteopenia and osteoporosis showed higher levels of mean spine T scores, Z scores, and BMD (Figure 2a–c), indicating improved bone health compared to the uninfected group. Similarly, the infected group also presented with increased femoral mean T scores, Z scores, and BMD (Figure 2d–f) compared to the uninfected individuals. It is to be noted that the uninfected and infected groups did not differ in their drug intake profiles for osteoporosis, but the duration of drug intake was significantly longer. Supplementary Figures S1 and S2 show that neither *T. gondii*-specific IgG levels nor IgG avidity exhibited a significant correlation with the mean spine, nor with the femoral T score, Z score, or BMD.

Furthermore, we segregated the total study subjects into osteopenia and osteoporosis groups and compared bone health indices between the infected and uninfected groups (Supplementary Figure S3). The infected osteopenic subjects exhibited significantly elevated mean spinal T score, Z score, and BMD (Supplementary Figure S3a–c). The femoral Z scores were significantly higher in the infected osteopenia group compared to the uninfected osteopenia group (Supplementary Figure S3e). However, infected and uninfected osteopenia patients had similar femoral T scores and BMD levels (Supplementary Figure S3d,f). In the osteoporosis group, infected participants had significantly higher spinal T and Z scores (Supplementary Figure S3g,h). There was no significant difference in spinal BMD between the infected and uninfected groups (Supplementary Figure S3i). Furthermore, the mean femoral T score, Z score, and BMD were significantly elevated in the infected group compared to the uninfected group (Supplementary Figure S3j–l). In addition, in both the osteopenia (*p* < 0.001) and osteoporosis (*p* < 0.004) groups, the infected subjects were significantly older than the uninfected subjects. This is in line with a previously conducted univariate analysis examining the relationship between *T. gondii* infection and age, which indicated a significant association (Exp (B) = 1.13, 95% CI (1.05–1.13), *p* < 0.001).

Different osteoporosis-related drugs (Denosumab, Romosozumab, Teriparatide, Bisphosphonate) were prescribed, as shown in Table 1. The duration of drug use was significantly longer in the infected group, but the age of treatment initiation was earlier in the uninfected group than in the infected group. In detail, the mean age of the uninfected group was 65.9, and their duration of treatment was 5.1 years, resulting in a mean age of starting treatment of 61 years. In contrast, the mean age of the infected group was 74.2, and their duration of treatment was 7.4 years, resulting in a mean age of starting treatment of 67 years. None of the osteoporosis drugs used exacerbated *T. gondii* infection.

**Figure 3:**
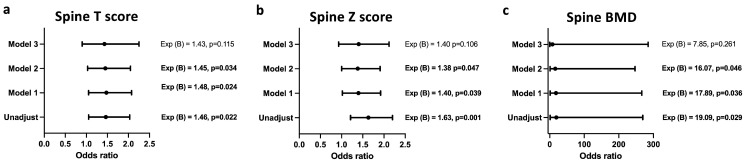
Regression model of the associations between spine DXA values and *T. gondii* infection. Association of *Toxoplasma* infection with spinal T score (**a**), Z score (**b**), and BMD (**c**). Unadjusted; Model 1: adjusted for age; Model 2: adjusted for age + creatinine + urea; Model 3: adjusted for age + creatinine + urea + duration of the therapy.

**Figure 4:**
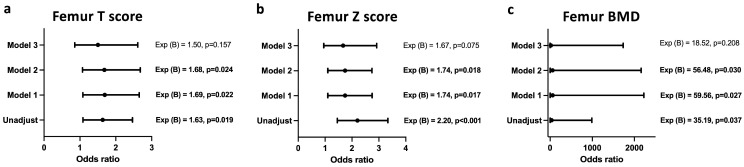
Regression model of the associations between femur DXA values and *T. gondii* infection. Association of *Toxoplasma* infection with femoral T score (**a**), Z score (**b**), and BMD (**c**). Unadjusted, Model 1: adjusted for age, Model 2: adjusted for age + creatinine + urea, Model 3: adjusted for age + creatinine + urea + duration of the therapy.

Figure 3 and Figure 4 show the logistic regression analysis between the bone mineral density indices of the spine and femur and the infection statuses of the study subjects. Infection with *T. gondii* was associated with increased odds of having a higher mean spine T score (Figure 3a), Z score (Figure 3b) and BMD (Figure 3c), even after adjusting for age, creatinine, and urea levels; however, after adjusting for the confounding factor—duration of therapy—the association of *T. gondii* infection with spinal T score, spinal Z score, and spinal BMD was lost.

A similar association was also observed between femoral T score (Figure 4a), Z score (Figure 4b), and BMD (Figure 4c) and the infection status after adjusting for age, creatinine, and urea levels. Upon adjustment for duration of therapy, the model lost its significance.

## 4. Discussion

Infections with the malaria-causing *Plasmodium* spp. parasites, which are closely related to *T. gondii* and belong in the apicomplexa group, have been shown to promote bone loss [23] and increase the susceptibility of Caucasians to osteoporosis [24]. Despite a high prevalence of *T. gondii* in Germany, it is surprising that there is a complete absence of studies addressing the question of whether and how an ongoing or past *T. gondii* infection could worsen or modulate the pathology and/or outcomes of osteoporosis. This question assumes significance in light of the fact that, during latent *T. gondii* infection, the parasite resides in the skeletal muscle tissue, and perturbations in the homeostasis of skeletal muscle have been shown to have deleterious consequences for the progression and severity of osteoporosis. With this background, we studied the association between osteopenia and osteoporosis and toxoplasmosis in a case–control study of patients visiting a tertiary care hospital.

The major finding of the study is that, in osteopenic and osteoporotic subjects positive for *Toxoplasma*, higher T scores of the spine and femur as well as higher bone mineral density of the spine and femur were observed. The univariate analysis revealed a significant association between age and *T. gondii* infection. This could be due to the fact that the *T. gondii*-infected subjects were significantly older compared to their uninfected counterparts. However, logistic regression analysis showed that the infected subjects had increased odds of having better mean T scores, Z scores, and mineral density of the spine and femur even after adjusting for potential confounders like age, urea, and creatinine levels. However, when the model was corrected for duration of therapy, the significance was lost. The infected patients also showed increased incidence of fractures, which may be due to their significantly older age and mobility issues. A report published by Zhu et al. (2022) [25] is the only other study available on the association between *T. gondii* infection and osteoporosis in human subjects. This study showed that women with *T. gondii* infection were at a higher risk of developing compound osteoporosis compared to those without infection. However, in case of male subjects with compound osteoporosis, such an association was not observed. Further, the study did not find an association of *T. gondii* with femoral or lumbar osteoporosis. Our findings differ from these observations in the following ways: (1) While the major focus of the study by Zhu et al. was on compound osteoporosis, we did not have a significant number of subjects with compound osteoporosis in our study group, and therefore, we cannot confirm this finding. (2) However, we found higher mean T scores and bone mineral density of the spine and femur in the individuals infected with *Toxoplasma,* suggesting a protective role of toxoplasmosis in subjects with osteoporosis and osteopenia, whereas the other report showed no association. However, it is to be emphasized that the increased duration of therapy had an influence on the increased femur and spine T and Z scores and BMD despite the increased incidence of fractures in the infected group. There could be several reasons for the discrepancies observed between the two reports. The differences in the study population could be attributed to the different ethnic groups involved. Another reason could be the fact that the study by Zhu et al. compared two groups—osteopenia vs. osteoporosis—and the prevalences of *Toxoplasma* were 10.44 and 19.37%, respectively, in the two groups. However, our study, consisting of 138 subjects, showed a higher prevalence of toxoplasmosis (46.4%), and we categorized the subjects as uninfected and infected with osteoporosis or osteopenia. It is to be noted that both groups had a similar distribution of osteopenic and osteoporosis subjects, and therefore, we do not expect that this could have skewed our study results.

On the other hand, our study findings are in agreement with the results of another in vitro study by Zhan et al. (2023) [26]. This report investigated the mechanisms through which the excretory and secretory products (ESPs) of *T. gondii* could affect the differentiation of bone marrow mesenchymal stem cells (BMSC) obtained from human samples. The findings of the study showcased that treatment of human BMSCs with ESPs of *T. gondii* promoted osteogenic differentiation. Furthermore, the report also demonstrated that ESPs of *T. gondii* could improve osteogenesis by modulating the Wnt/β-catenin signaling pathway as well as aerobic glycolysis in human BMSCs.

There are several limitations of the study. This was a case–control study, and no causal associations between bone health parameters and toxoplasmosis could be drawn. The study also lacked a control group of subjects with normal bone health that were infected and uninfected. Due to the heterogeneity and small cohort size of patients with various exclusion criteria, we could not study *Toxoplasma* infection in highly immune-suppressed patients. Further, this was a hospital-based study; therefore, the findings of the study cannot be extrapolated to the general population. However, all of these points are great ideas for future (multicenter) studies and should be considered in the study design.

This is the first study from Germany to show an association between toxoplasmosis and bone density in osteopenia and osteoporosis patients, opening up a new field of research into how *T. gondii* affects bone health.

## Figures and Tables

**Table 1:** Clinical and biochemical characteristics of the study participants.

Parameters	Uninfected (*n* = 74)	Infected (*n* = 64)	*p*
Age (years)	65.9 ± 10.2	74.2 ± 9.6	**<0.001**
Gender (M/F) (*n*)	14/60	8/56	0.356
25OH vitamin D (ng/mL)	45.6 ± 17.1	38.6 ± 17.1	**0.018**
Alkaline phosphatase (U/L)	75.8 ± 30.2	80.8 ± 31.9	0.348
Alanine-aminotransferase (U/L)	22.1 ± 12.8	22.8 ± 22.3	0.801
Aspartate-aminotransferase (U/L)	24.6 ± 6.4	30.0 ± 38.6	0.239
Creatinine kinase (U/L)	93.4 ± 53.6	127.2 ± 172.7	0.114
Gamma-glutamyltransferase (U/L)	30.8 ± 33.7	32.8 ± 29.0	0.723
Bone alkaline phosphatase (μg/L)	16.3 ± 8.1	18.6 ± 9.4	0.138
Lactatedehydrogenase (U/L)	213.5 ± 36.5	227.4 ± 48.59	0.059
Thyroid-stimulating hormone (μU/mL)	1.5 ± 1.1	1.7 ± 2.1	0.354
CRP (mg/L)	4.7 ± 9.1	9.8 ± 18.3	0.056
Calcium (mmol/L)	2.4 ± 0.1	2.3 ± 0.1	0.207
Magnesium (mmol/L)	0.84 ± 0.06	0.83 ± 0.08	0.570
Sodium (mmol/L)	139.1 ± 2.9	139.2 ± 3.4	0.720
Potassium (mmol/L)	4.5 ± 0.4	4.5 ± 0.5	0.363
Inorganic phosphate (mmol/L)	1.13 ± 0.2	1.17 ± 0.2	0.277
Iron (μg/dL)	91.0 ± 35.2	91.6 ± 38.7	0.937
Ferritin (ng/mL)	133.6 ± 104.0	158.3 ± 147.3	0.263
Total bilirubin (mg/dL)	0.46 ± 0.25	0.51 ± 0.34	0.333
Urea (mg/dL)	30.3 ± 8.2	34.6 ± 12.5	**0.018**
Creatinine (mL/min)	0.77 ± 0.19	0.86 ± 0.31	**0.047**
Immunoglobulin A (g/L)	2.4 ± 1.2	2.4 ± 1.0	0.932
Immunoglobulin G (g/L)	10.0 ± 2.4	10.2 ± 2.5	0.715
Immunoglobulin M (g/L)	1.1 ± 0.8	0.9 ± 0.6	0.296
Leucocytes (G/L)	7.4 ± 2.1	7.4 ± 1.9	0.984
Rheumatoid arthritis (%)	12.2	10.9	0.757
COPD (%)	10.8	6.25	0.307
Crohn’s disease (%)	2.7	1.5	0.622
Fracture history (%)	55.4	78.1	**0.006**
Duration of the therapy (years)	5.1 ± 3.6	7.4 ± 5.2	**0.019**
Lowest T score	−3.07 ± 1.02	−2.75 ± 1.19	0.106
Latest best T score	−2.30 ± 0.88	−2.06 ± 0.79	0.172
Drug (present/past history)			
Denosumab (%)	62.8	70.4	0.458
Romosozumab (%)	4.3	13.1	0.112
Teriparatid (%)	12.8	16.3	0.624
Bisphosphonate (%)	24.2	37.7	0.128
*T. gondii*-specific IgG levels (IU/mL)	-	65.3 ± 72.9	N/A
*T. gondii*-specific avidity	-	0.52 ± 0.13	N/A

Significant *p*-values are highlighted in bold.

## Data Availability

The original contributions presented in the study are included in the article/Supplementary Materials. Further inquiries can be directed to the corresponding author.

## Supplementary Materials

The following supporting information can be downloaded at: https://www.mdpi.com/article/10.3390/biomedicines12071400/s1, Figure S1: Correlation analysis of femur and spine DXA values with *T. gondii*-specific IgG levels. Correlation of *T. gondii*-specific IgG between spinal T score (a), Z score (b), and BMD (c). Correlation of *T. gondii*-specific IgG with femoral T score (d), Z score (e), and BMD (f); Figure S2: Correlation analysis of femur and spine DXA values with *T. gondii*-specific IgG avidity. Correlation of *T. gondii*-specific IgG avidity between spinal T score (a), Z score (b), and BMD (c). Correlation of *T. gondii*-specific IgG avidity with femoral T score (d), Z score (e), and BMD (f); Figure S3: Bone health indices in the uninfected and infected osteopenia and osteoporosis cohorts. For the osteopenia cohort, the violin plots depict the mean spinal T score (a), Z score (b), and BMD (c) and the mean femoral T score (d), Z score (e), and BMD (f). For the osteoporosis cohort, the violin plots show the mean spinal T score (g), Z score (h), and BMD (i) and the mean femoral T score (j), Z score (k), and BMD (l). Statistical significance was determined.
