# Supplementary material for: Seroprevalence and Association of Toxoplasma gondii with Bone Health in a Cohort of Osteopenia and Osteoporosis Patients
Source: Biomedicines. 2024 Jun 24;12(7):1400. doi: 10.3390/biomedicines12071400 (PMC11274202; doi:10.3390/biomedicines12071400)
Supplement: Supplementary file 1 [file biomedicines-12-01400-s001.zip › biomedicines-3026215-supplementary.pdf]

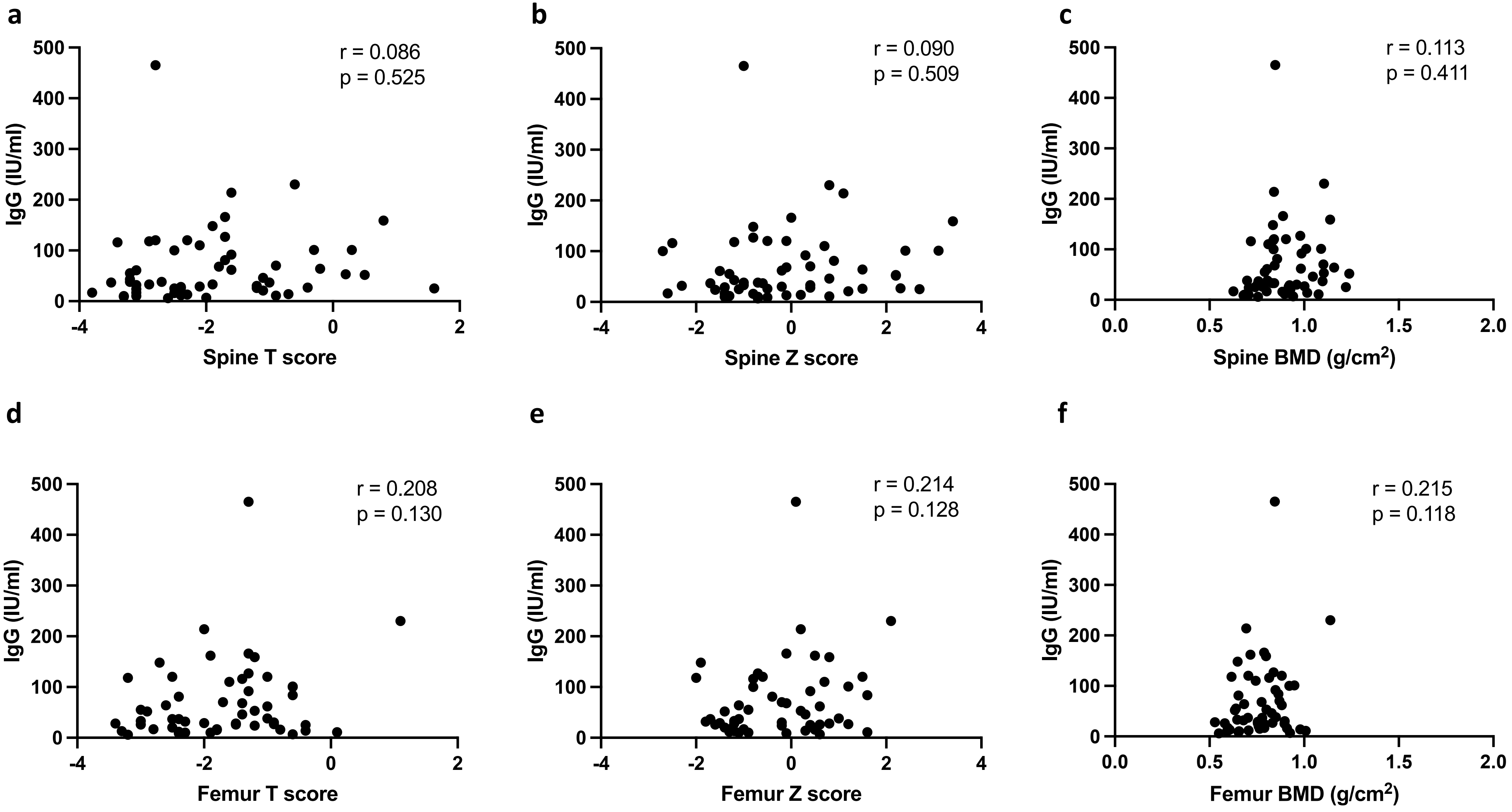

Figure S1: Correlation analysis of femur and spine DXA values with T. gondii-specific IgG levels. Correlation of T. gondii-specific IgG between spinal T score (a), Z score (b), and BMD (c). Correlation of T. gondii-specific IgG with femoral T score (d), Z score (e), and BMD (f).

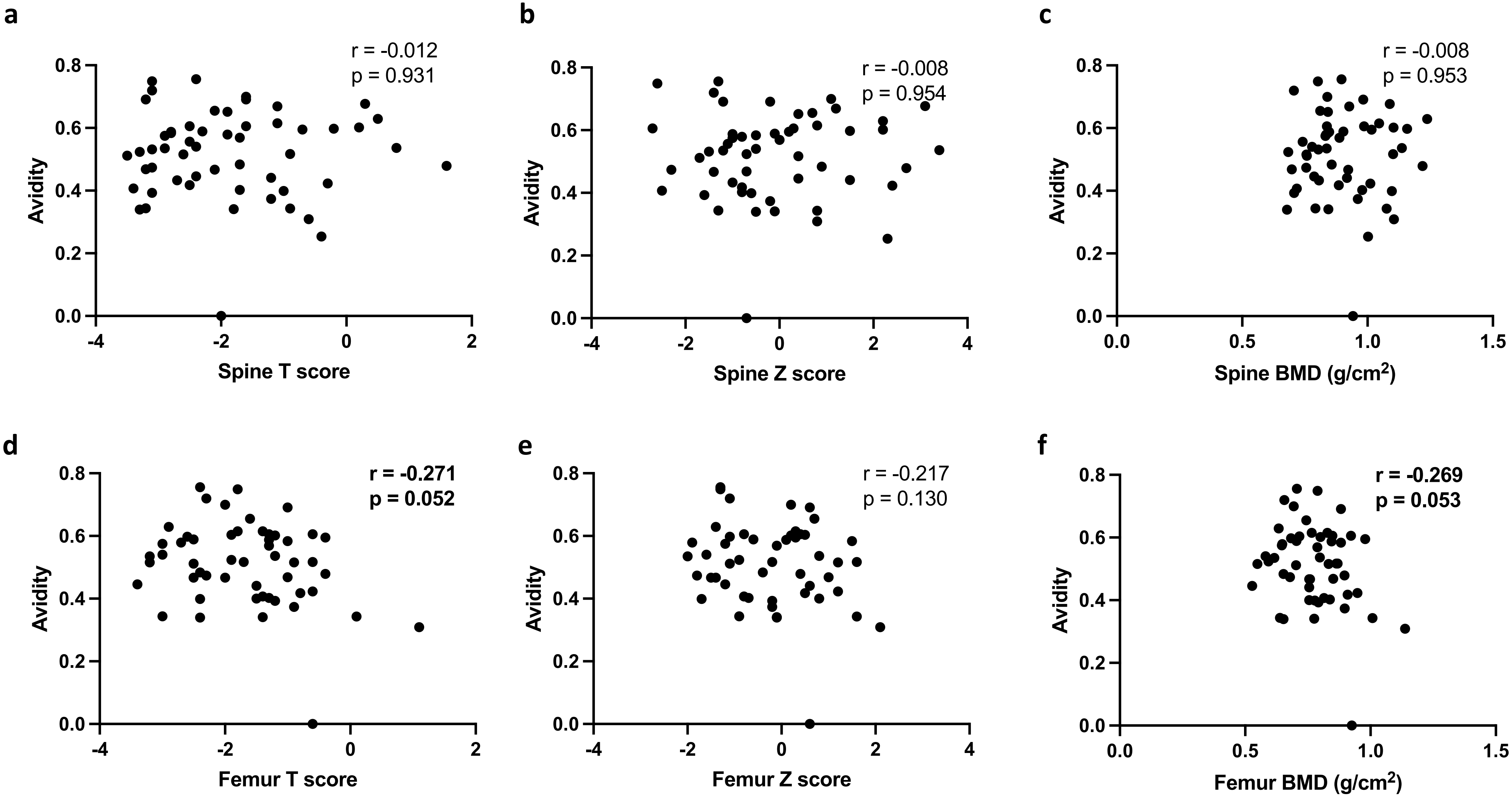

Figure S2: Correlation analysis of femur and spine DXA values with T. gondii-specific IgG avidity. Correlation of T. gondii-specific IgG avidity between spinal T score (a), Z score (b), and BMD (c). Correlation of T. gondii-specific IgG avidity with femoral T score (d), Z score (e), and BMD (f).

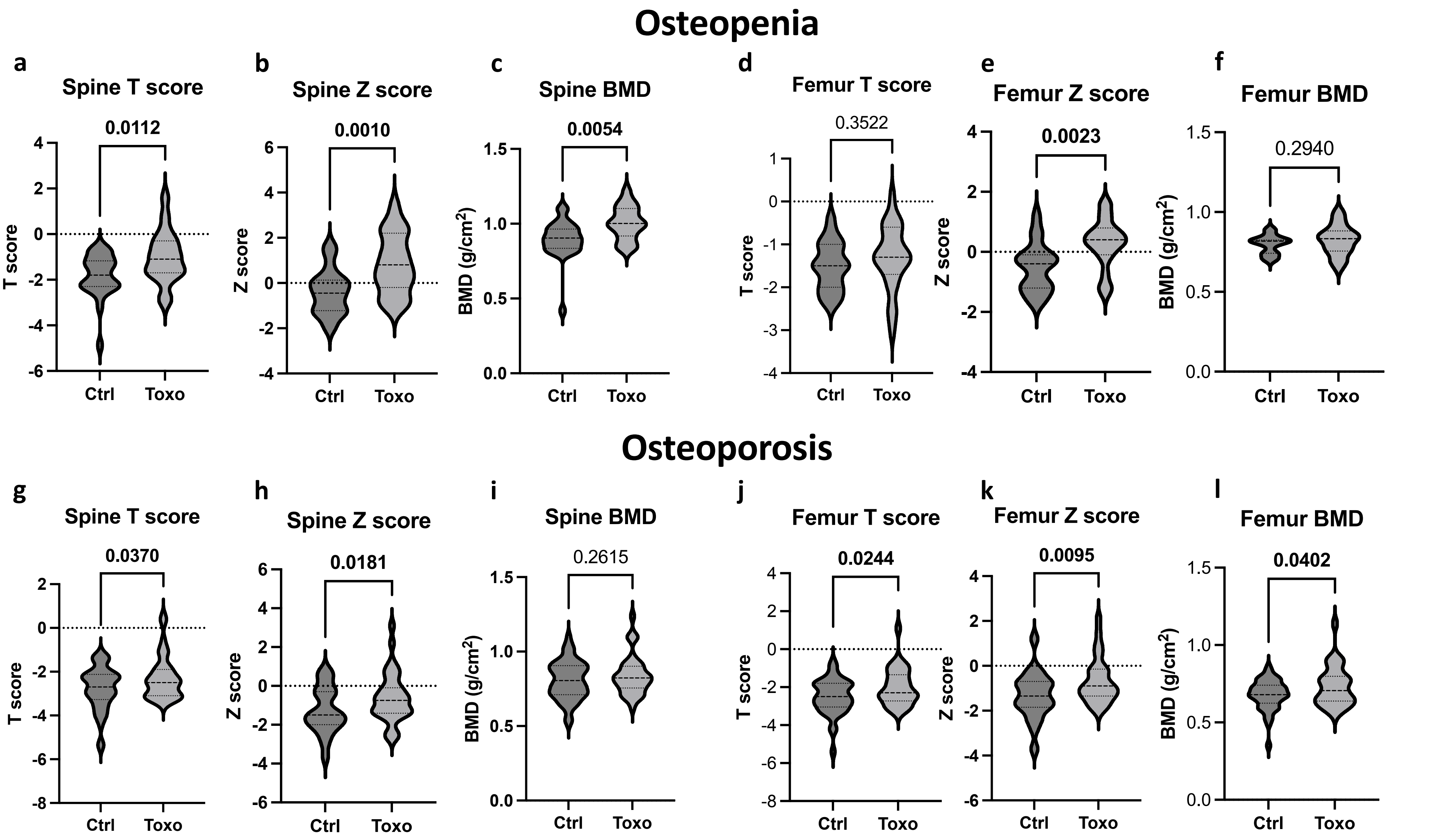

Figure S3: Bone health indices in the uninfected and infected osteopenia and osteoporosis cohorts. For the osteopenia cohort, the violin plots depict the mean spinal T score (a), Z score (b), and BMD (c) and the mean femoral T score (d), Z score (e), and BMD (f). For the osteoporosis cohort, the violin plots show the mean spinal T score (g), Z score (h), and BMD (i) and the mean femoral T score (j), Z score (k), and BMD (l). Statistical significance was determined.
